# Health workforce development and retention in Guinea: a policy analysis post-Ebola

**DOI:** 10.1186/s12960-019-0400-6

**Published:** 2019-08-05

**Authors:** Remco van de Pas, Delphin Kolie, Alexandre Delamou, Wim Van Damme

**Affiliations:** 1 Department of Public Health, Institute of Tropical Medicine Antwerp, Nationalestraat 155, 2000 Antwerp, Belgium; 2 Centre National de Formation et de Recherche en Santé Rurale de Maferinyah, Ministère de la Santé, 2640 Forécariah, Guinea; 3 Department of Health Ethics and Society, Faculty of Health Medicine and Life Sciences, Maastricht University, P.O. Box 616, 6200 MD Maastricht, The Netherlands; 4 Department of Public Health, Gamal Abdel Nasser University, Conakry, Guinea

**Keywords:** Ebola virus disease, Health systems reform, Health workforce retention, Human Resources for Health Governance, Decentralization, Health workforce finance

## Abstract

**Background:**

The state of the Guinean health workforce is one of the country’s bottlenecks in advancing health outcomes. The impact of the 2014–2015 Ebola virus disease outbreak and resulting international attention has provided a policy window to invest in the workforce and reform the health system. This research constitutes a baseline study on the health workforce situation, professional education, and retention policies in Guinea. The study was conducted to inform capacity development as part of a scientific collaboration between Belgian and Guinean health institutes aiming to strengthen public health systems and health workforce development. It provides initial recommendations to the Guinean government and key actors.

**Methodology:**

The conceptual framework for this study is inspired by Gilson and Walt’s health policy triangle. The research consists of a mixed-methods approach with documents and data collected at the national, regional, and district levels between October 2016 and March 2017. Interviews were conducted with 57 resource persons from the Ministry of Health, other ministries, district health authorities, health centers and hospitals, health training institutions, health workers, community leaders, NGO representatives, and development partners. Quantitative data included figures obtained from seven health professionals’ schools in each administrative region of Guinea. A quantitative analysis was conducted to determine the professional graduate trends by year and type of personnel. This provided for a picture of the pool of professional graduates available in the regions in relation to the actual employment possibilities in rural areas. The districts of Forecariah and Yomou were chosen as the main study sites.

**Results:**

Limited recruitment and a relative overproduction of medical doctors and nurse assistants have led to unemployment of health personnel. There was a mismatch between the number of civil servants administratively deployed and those actually present at their health posts. Participants argued for decentralization of health workforce management and financing. Collaboration between government actors and development partners is required to anticipate problems with the policy implementation of new health workers’ deployment in rural areas. Further privatization of health education has to meet health needs and labor market dynamics.

## Background

The state of the Guinean health workforce (HWF) is one of the country’s bottlenecks in advancing health outcomes [1]. There has been a decade-long underinvestment with limited public recruitment and a workforce dominated by medical doctors [2, 3]. In 2014, a workforce projection study found that maternal and neonatal health services require particular attention. The main shortage is in skilled birth attendance where only 18% of needs are met [4]. Although there is a relative oversupply of general practitioners and nurse assistants (ATS), many of them work in the private, informal sector. There are considerable variations in the distribution of health personnel given the human resources for health (HRH) needs and HRH supplies between rural and urban areas [4]. While overall HRH needs for maternal and neonatal health services were projected to increase by 22% between 2014 and 2024, the supply was projected to decline by 15% under existing recruitment patterns [4].

**Fig. 1:**
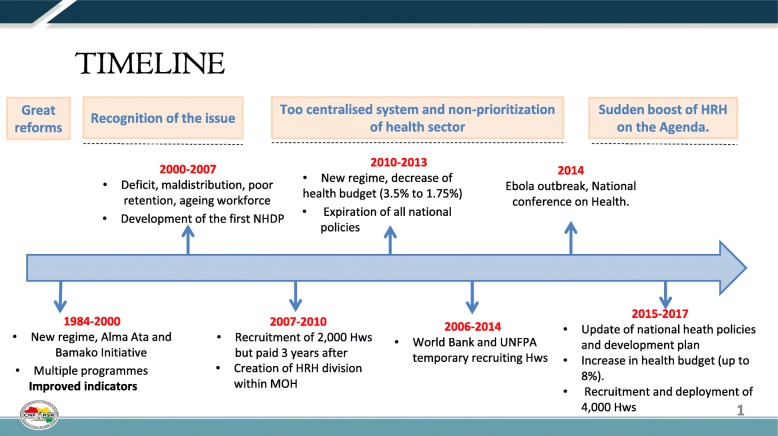
Evolution of HRH policy in Guinea post-Ebola

The Ebola virus disease (EVD) outbreak of 2014–2015 facilitated international finance and humanitarian assistance and spurred the United Nations (UN) Security Council to create the first ever UN mission for a public health emergency [5]. In the wake of EVD, there has been much debate and proposals for global health governance reforms by the World Health Organization (WHO) and other institutions to address future epidemics and build resilient health systems. Authors have suggested that the EVD outbreak could be a transformative moment in recognizing that there are shared responsibilities by governments in strengthening health systems [6]. The workforce is now considered a crucial pillar for global health security and has been included in the Sustainable Development Goals (SDG)^1^ [7, 8]. The WHO’s Global Strategy on HRH: Workforce 2030 includes a new estimate of the workforce density required to meet the SDG^2^ [9]. This figure is ten times the proportion of HWF currently employed by the Guinean public sector [1]. A UN commission has also provided a report and action plan on the importance of health employment for economic growth [10]. In Guinea itself, the impact of the EVD outbreak and resulting international attention has provided a policy window to invest in the workforce and reform the health system after many years of stagnation (see Fig. 1). Indeed, the government implemented a health system recovery and resilience strategy with the intention to recruit 6000 staff from 2016 to 2018 and increase their salaries by 40% [11]. In 2016, the Ministry of public services recruited 3802 health workers (HWs) who signed a 5-year contract committing to work in rural areas and were deployed in March 2017 [12]. International development partners have provided much support to further strengthen Guinea’s health system, including 25 million Euros from the European Union in 2015, provided that the government expands its fiscal expenditure on health [13]. By 2017, the government increased the total health expenditure from 4 to 8% [14].

Meanwhile, the government has planned to reform HRH policy and management functions (Table 1).

**Table 1:** Reforms by the Guinean government in HW policy and management functions [1]

• To develop a national strategy to retain staff in remote areas via decentralized training and recruitment. • Strengthen the institutional framework for HRH recruitment and management performance. • Re-concentrate initial formation of health workers to improve the quality of care; • Strengthen the capacity of health education institutions through inter-sectoral collaboration. • Strengthen the skills of personnel in terms of quality of care and health management. • Establish a strategy to develop and motivate a Community Health Workforce [14]. • Reform of HRH policy and management is part of institutional developments in Guinea in which policy dialogue and health coordination structures have been established over the last years [15].	

Complementarily, the government has strengthened essential public health functions such as epidemiological surveillance by creating regional alert and response teams as well as a National Health and Safety Authority (ANSS) [15, 16]. Guinea’s Ministry of Health (MoH) has announced plans to invest in 11 000 new HWF jobs over the next decade [17].

This research constitutes a baseline study to inform scientific and educational capacity development as part of a scientific collaboration between the Institute of Tropical Medicine Antwerp (ITM) and the Centre National de Formation et de Recherche en Santé Rurale (CNFRSR), Maferinyah, aiming to strengthen public health systems and HWF development in Guinea. The study advances academic debates on how to further HWF development for resilient health systems in fragile contexts [18]. The objectives and analysis are informed by a framework for HWF labor market dynamics [19]. The study aimed to assess whether a regional pool of qualified health workers could be a basis for attracting unemployed HW to be recruited to work in rural areas. It provides initial recommendations to the Guinean government and key actors in improving education, retention, and sustainability of staff recruited to work in rural areas. Consecutive studies will analyze the policy process, implementation, and health systems’ impact of employing HWF in remote areas. Two main research objectives have been identified:

1. To assess the dynamics of HWF retention in rural areas in the post-Ebola period.
2. To assess the availability of HWF education in relation to the labor market supply.

This study was conducted *after* the policy decision to employ HWs in rural areas, but *before* the actual recruitment and deployment in 2017. The study intends to provide policy guidance facilitating the actual retention of HWF. This article is a short version of a research report presented to the MoH and other relevant actors in a workshop in September 2017 [20].

**Fig. 2:**
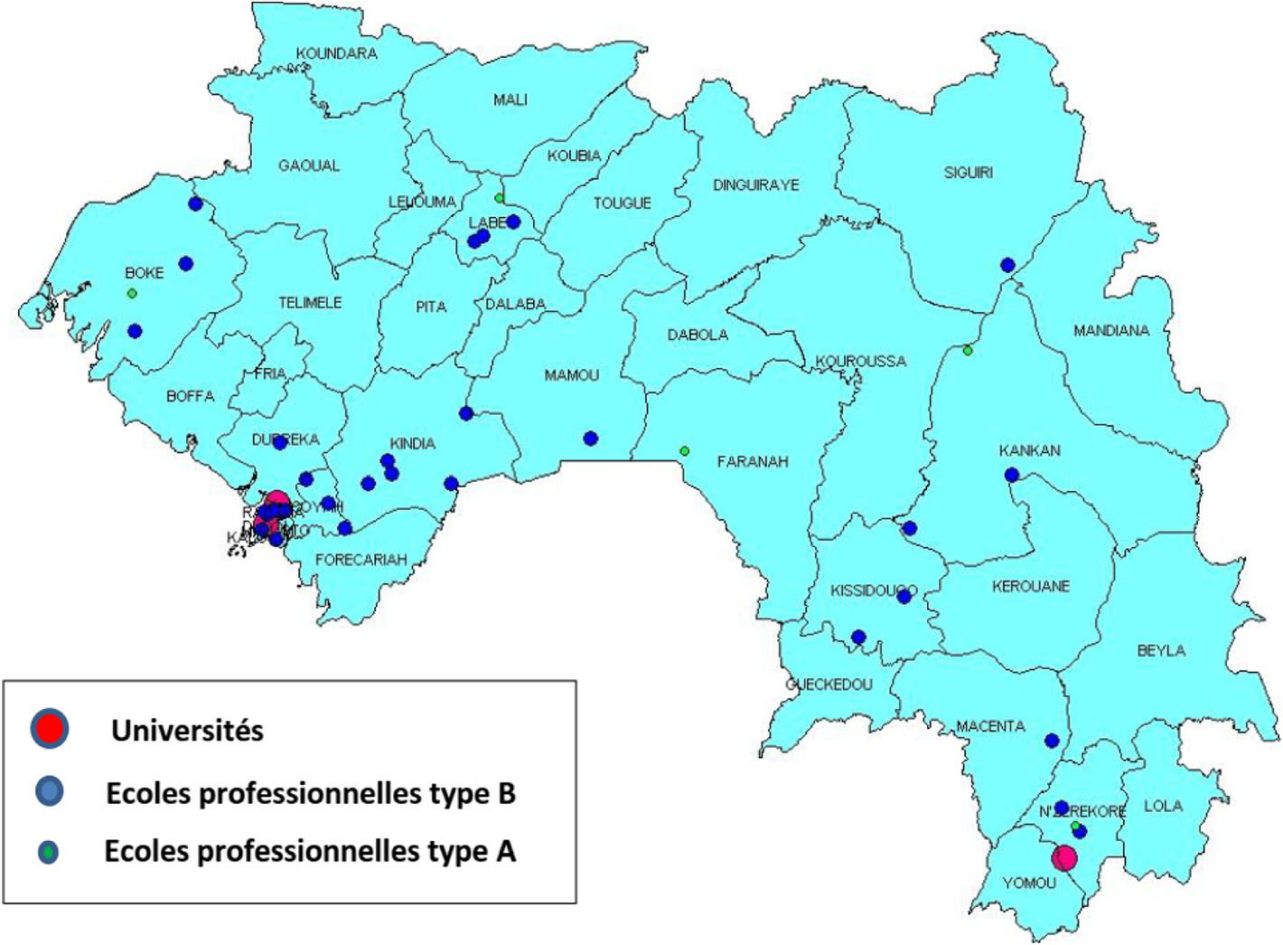
Geographic distribution of health training institutions in Guinea, April 2017

## Methodology

The conceptual framework for this study follows Gilson and Walt’s health policy triangle which indicates how different actors (individuals, government, and national/international organizations) interact to influence formulation, planning, implementation, and evaluation of health policies. This model also helps to assess perceptions, processes, and complexities of established strategies [21]. In this study, the policy triangle has been applied prospectively to anticipate the formulation and implementation of the new deployment policy. It provides for understanding the processes through which influence is played out and how actors and the political situation during and after EVD outbreak shape and implement the new HWF policy [21]. The different elements of the health policy triangle provide the structure for the “Results” and “Discussion” sections. These follow the interrelated components of the policy triangle: situational context, policy content, process implementation, and actors involved. This study is a mixed-methods approach with data collected at the national, regional, and district levels between October 2016 and March 2017. Interviews were conducted with 57 key actors involved at multiple levels of capacity development, training, and management of the HWF, both in and outside government. Quantitative data were collected on the aggregated pool of qualified HW available as well as absenteeism and retention rates of HW at the district level.

Table 2 depicts an overview of the study participants: respondents were purposefully selected and, via snowball sampling, additional key actors were included [22].

**Table 2:** Number of interviews conducted per groups of participants

Groups of participants	Numbers interviewed
Developments partners/donors, international local NGO representatives	12
Officials of the Ministry of Health (central, regional and district authorities)	13
Officials of the Ministry of Education and Training Institutions	08
Officials of the Ministry of Public/Civil Services	02
Officials of the Ministry of Decentralization (regional and local authorities)	05
Health workforce (civil servants working as health-facility managers or caregivers, contractors, and volunteers)	17
Total	57

The health districts of Forecariah and Yomou were chosen as the main study sites and represent two very different rural contexts. This first study focused on rural areas only given the gap between HWF needs and supplies between Conakry and the rest of the country [4]. Given feasibility and timeframe, only two study sites were selected at this stage.

Forecariah is in lower Guinea and well accessible by road in 2 h from Conakry and provides for personal and market mobility. Yomou is situated over thousand kilometers from Conakry in the Guinean forested region close to the Liberian border. There are fewer government investments in infrastructure, education, and services than lower Guinea. The HWF situation in Forecariah is less deficient than in Yomou, but the former has been severely affected by EVD, with 433 cases identified and 10 HWs infected. In Yomou, only 10 cases were identified.

**Fig. 3:**
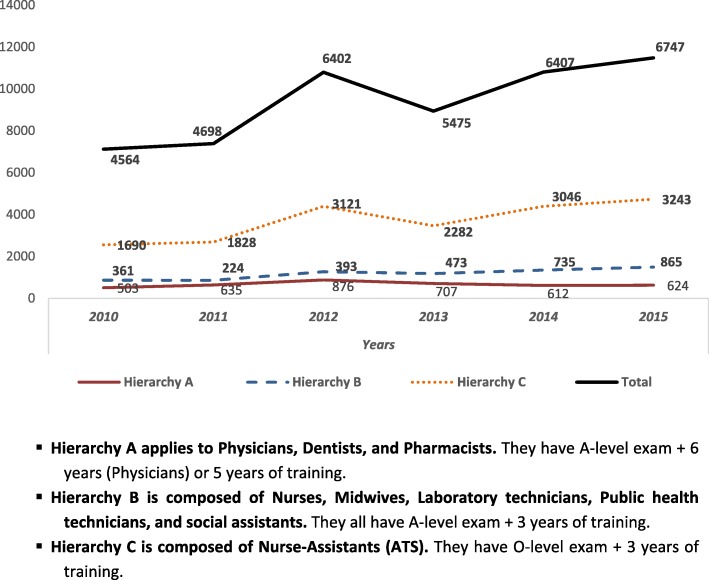
Distribution of graduates from 2010 to 2015 by staff category. Hierarchy A applies to physicians, dentists, and pharmacists. They have A-level exam + 6 years (physicians) or 5 years of training. Hierarchy B is composed of nurses, midwives, laboratory technicians, public health technicians, and social assistants. They all have A-level exam + 3 years of training. Hierarchy C is composed of nurse assistants (ATS). They have O-level exam + 3 years of training

Directors of the seven health professionals’ schools in each administrative region of Guinea were interviewed to obtain data on the HWs trained during the preceding 5 years and to assess whether these nursing/midwifery schools were functioning in the post-Ebola period (Fig. 2). Data on type and number of graduates per year were retrieved from the seven school registries. An overview was generated to determine the professional graduate trends by year and type of personnel, which provide insights on the decentralized supply of HWs in the regions (Fig. 3).

Qualitative data were collected through semi-structured interviews based on interview guides that were piloted and improved afterwards. This included questions on how aggregated professional graduate trends (supply) match with labor market needs, HWF retention dynamics in the rural districts, and recruitment policies. The interviews were then fully transcribed. The two main researchers independently coded and analyzed the results following a coding grid that corresponded to different elements of the health policy triangle. Quantitative data were collected using a pre-developed form that was amended after a pilot phase. At district levels, HWF registers were consulted and absenteeism rates were calculated using the civil servants’ registry and comparing these with data from HWF being present as reported by district health authorities.^3^

All participants received an information sheet and provided a signed consent to be included in the study. Study participants and key actors could provide feedback on the draft report during a research workshop in July 2017. The study was approved by the national research ethics committee in Guinea^4^.

## Results

The results are structured according to the two main research objectives. The first objective on HWF retention is presented along the 4 interrelated components of the health policy triangle. The quantitative results on HWF education are presented afterward.

### The dynamics of HWF retention

#### HWF Situation and contextual factors

An absolute shortage of HWF in both districts according to global HRH policy guidelines^5^ was observed. This shortage was more or less similar between Yomou and Forecariah. In total, 289 HWs were registered in Forecariah and 135 in Yomou. This corresponds with a ratio of 1.2 available HWs per 1000 persons in each of the districts (Table 3).

**Table 3:** Health workforce profiles and distribution in Forecariah and Yomou districts, Guinea, December 2016

	Forecariah number (%)	Yomou number (%)
Health workforce per status		
Civil servants*	83 (29)	43 (32)
Contractual/volunteers	206 (71)	92 (68)
Total	289 (100)	135 (100)
Health workforce distribution per status and categories in rural areas		
Civil servants		
Type A	5 (6)	0 (0)
Type B	4 (5)	7 (16)
Type C	14 (17)	9 (20)
Informal	0 (0)	0 (0)
Sub-total	23 (27)	16 (37)
Contractual/volunteers		
Type A	0 (0)	1 (1)
Type B	22 (11)	12 (13)
Type C	31 (15)	25 (27)
Informal	99 (48)	36 (39)
Sub-total	152 (74)	74 (80)
Health workforce distribution per status and categories in the urban center of the district		
Civil servants		
Type A	12 (14)	5 (12)
Type B	19 (23)	11 (26)
Type C	30 (36)	11 (26)
Informal	0 (0)	0 (0)
Sub-total	61 (73)	27 (63)
Contractual/volunteers		
Type A	7 (3)	0 (0)
Type B	17 (8)	0 (0)
Type C	18 (9)	18 (20)
Informal	12 (6)	0 (0)
Sub-total	54 (26)	18 (20)

*83 health workers were present out of the 202 posted in Forecariah. In Yomou, this was 43 out of 111 health workers

We also found an important mismatch (absenteeism) between the number of HWs deployed (according to the civil servant registry) and those actively working in the district (according to the district authorities). In fact, of 202 civil servants posted in Forecariah, only 83 were present at their post (absenteeism rate 41%). This absenteeism rate was 39% (43 present of 111 posted) in Yomou. Some participants argued that most of the healthcare tasks were actually provided by local contractors or volunteers, who hope to be prioritized during civil servant recruitment processes.

Participants in the districts mentioned that the top-down model of civil servant recruitment does not favor retention in rural areas. Participants reported that the staff, purposefully recruited from the capital, was parachuted in underserved areas for just a few months to benefit their civil servant salary, while volunteers or contractors working for decades in the municipalities had fewer chances of being recruited.

We have recommended the central level to recruit people already working with us as volunteers or contractors for years but they prefer recruiting those who are prepared for anything except staying in rural areas (IDI 15, member district health office).

Furthermore, the available civil servants in both districts were unequally distributed as depicted in Table 3. Most of them worked in the urban conglomerations either at the district management levels, district hospitals, or urban health centers. In remote areas, temporary contracted HWs and volunteers (graduated HW without formal employment) were the most represented. Additionally, there was also an imbalance in the distribution of HWs according to their professional categories. Doctors, pharmacist, and dentists (type A), and nurses, laboratory technicians, and midwives (type B) were mainly found in urban areas while assistant nurses (type C) and informal community health workers occupied facilities in rural areas.

HWF aging is another highlighted challenge; many HWs will retire in the coming decade [4]. The main point emerging at local levels is that the state should accord sufficient resources and engage with development partners to favor decentralization of its recruitment, deployment, and payment policies. At the central level, participants identified three major issues: first, the state is solely responsible for its employees and should not have support from partners regarding their salaries; second, several donors are, in principle, committed to supporting the state but this assistance has been arriving slowly;

The EU wants to support the government in this process but the funding has not yet been received. The EU requires an indication of further deconcentration and decentralization of personnel management within the country. (IDI 21, development partner)

Third, partners committed to supporting the government in sustainable health system reforms have themselves recruited HWs into projects that they support.

### HRH policy on recruitment and deployment

Participants reported that in the post-EVD period, staffing shortfall was temporarily solved by ANSS and the United Nations Population Fund (UNFPA) maternal health project contracting and deploying HWs. District health managers were worried about the contracts of ANSS HWs ending soon. At the district level, respondents suggested that contracted HWs and interns from the health centers are prioritized in the new recruitment; however, this should also be followed by an administrative decentralization of HWF management. HWF supervision was considered inefficient with limited decision space at the district levels. Respondents also argued for annual recruitments rather than the currently practiced 5-yearly recruitments.

It was also suggested that the state take gender issues into account when deploying staff since married women may prefer to work in urban areas or near their husbands. Respondents mentioned that recruitment from outside the region had negative impacts mainly concerning responsibilities and trust between staff members.

Participants provided policy propositions for improved HWF retention. This includes local recruitment, strengthening supervision, allocation of wages by the local administration, including community overview, adopting career plans and rotation schemes for staff, developing medical specialist positions in rural hospitals, improving living and working conditions, and creating incentives (financial) and a particular status for health staff working in rural areas. One participant confirmed that there are ongoing reflections to initiate real reform of the national human resources policy.

The state needs to consider creating a local public service in every region of the country. This service could be directly under the authority of the regional governor and would be responsible for recruiting state officials, including health professionals. (IDI 14, government officer)

### Anticipated process implementation of the HWF policy

Some local actors reported that central public administration officials would be the first to undermine a new transparent retention policy. They would do so by favoring persons during the deployment process or by refusing to adopt punitive measures against those who would not stay in their job positions. Many HWs recruited during the last round did not comply with their contract of engagement.

Approximately 40% of participants were in favor of a 5-year service contract working in rural areas and some were ready to be deployed to such areas. Respondents believe that new staff deployment will have a positive impact on improving coverage and provision of care. Nevertheless, others said that the lack of state transparency in HWF recruitment is a demotivating factor in seeking employment in health services in rural areas.

In other countries, people line up to work in rural areas as it allows them to access training grants but at ours, those who refuse to go to the interior are the same ones who could finalize their studies abroad or who are promoted to positions of responsibility? (IDI 30, health worker)

Although all respondents appreciated the new recruitment of HWs, many had a negative perception of its organization and reported low recruitment rates of specialists. Respondents argued that the state should pay more attention to collaboration with the private sector which could be a real driver of employment and growth for the country.

### Actors, values, positions, and collaborations on HWF policy

Educational and ministerial actors who are responsible for the training, recruitment, and central management of HWF confirmed that the MoH was not playing its full role in HWF management. According to them, there is an overproduction of less-qualified HWs in the country. Additionally, their training curriculum does not follow the needs and priorities set by the MoH. Respondents reported that the MoH, which deploys HWs, collaborates poorly with the Ministry of Finance (MoF) regarding their payment.

We do not control the training of health workforce, let alone its recruitment or salary. All these related services report to their line ministries and not to the Ministry of Health. (IDI 23, government officer)

Central actors and respondents from a non-governmental organization (NGO) and a multilateral organization were of the opinion that the MoH’s main task is the supervision of the HWF. They argued that poor coordination between ministries negatively impacted the sustainability of health gains. Moreover, MoH respondents had doubts about the functioning of an inter-ministerial HWF committee as a consultative platform. The ministry has proposed to transform the human resources division and create an HWF directorate but respondents argued that such institutional development should be accompanied by the allocation of more financial resources to strengthen capacities and staff supervision.

Representatives of international organizations reported that their institutions would recruit HWs in their areas of intervention and thereby accompany the state in implementing the health system’s recovery and resilience plan. The development partners would be distributed between several regions to allow coverage of the whole country but also to evaluate the impact on the improvement of the system in each actor’s area of intervention.

We are recruiting health workforce with the highest criteria to run our health projects. The contract we have with this health workforce is already perceived by the state as a hiring contract in the public service. The state does not respect its commitments and this poses a problem for maintaining this staff in the project areas. (IDI 24, development partner)

There has been progress in improving community-based surveillance through support from the International Organization for Migration. Additionally, both Forecariah and Yomou districts benefited from staff being recruited and contracted by the ANSS. The district hospital management and health authorities reported good collaboration between them and ANSS staff. Although these managers appreciated the availability and motivation of ANSS staff, they entrusted also that this staff lacked experience in monitoring activities. ANSS employees, meanwhile, reported difficulties in integrating immunization campaign supervision activities at the district level. They also struggled in participating in regular training activities organized by the district health team. This integration challenge is partly due to parallel financial and management systems.

### HWF education and its relation to the labor market supply

The results in this section provide a quantitative overview of the HWF education in Guinea. Figure 2 indicates a mal-distribution of health training institutions across the country. Most are located in Lower Guinea, particularly in Conakry. Much of Middle and Upper Guinea lacks health training institutions. This disparity mainly concerns universities and professional schools (type B) which train state nurses, midwives, laboratory and public health technicians, and social assistants.

Type A vocational schools that train ATS are more or less well-distributed throughout the country, although some type B vocational schools also train ATS. There are four higher education institutions (universities) in Guinea: three in Conakry (two are private) and one in N'zérékoré.

Figure 3 shows an upward trend in the number of graduates (total 15 000 HWs) trained in Guinea between 2010 and 2015 and that ATS, state nurses, and midwives were the largest professional groups trained. Almost all professional categories (except ATS) were trained in lower Guinea, amounting to nearly 44% of the HWF trained over the last 5 years.

**Fig. 4:**
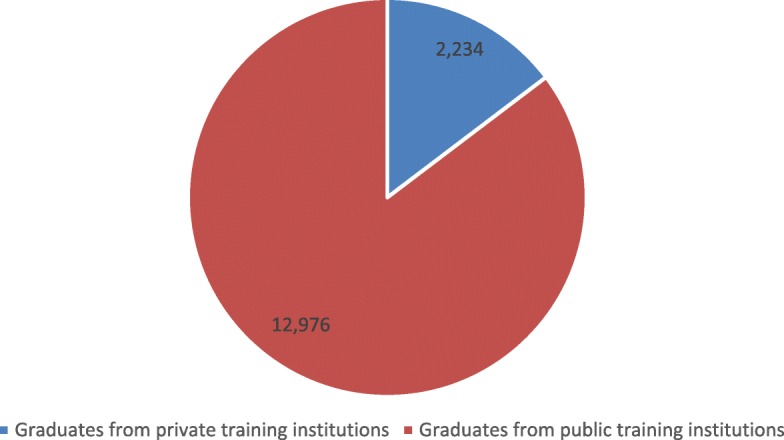
Distribution of graduates from 2010 to 2015 by type of institution (private or public)

Figure 4 shows the importance of private institutions in the training of certain professional categories between 2010 and 2015. Midwives, state nurses, and public health technicians were primarily trained in private institutions. Doctors, pharmacists, dentists, social assistants, and ATS were exclusively trained in public institutions.

## Discussion

The results provide an account of the challenges and possible solutions to improve HWF development in Guinea. The discussion builds on the results through analyzing different elements of the health policy triangle, followed by an analysis of the educational data, and finally some initial HWF policy, and governance recommendations and implications.

### HRH situation and contextual factors

It is challenging to employ and retain HWF in rural districts. The results indicate that the economic development situation makes it difficult for HWs to establish themselves permanently leading them to be often absent from their workplace and district where deployed. They can only rely on a limited civil servant salary and there is less economic demand for additional private health services than in Conakry. Women HWs, generally about 60–70% of the global workforce, face particular challenges. International evidence points to systemic gender discrimination and inequalities in pre-service and in-service health education and (rural) employment [23]. While not specially addressed in this study, it is of importance to include a gender and equity analysis in follow-up studies tracing HWF policy implementation in Guinea [24].

Respondents mentioned the need to be able to recruit and manage the HWF locally. Since salaries are paid at the central level, district-level health-team managers have no leverage to incentivize or sanction HWs’ efforts. In general, it is crucial to find a good balance in dividing HWF governance responsibilities across central, regional, and district institutions [25]. Ongoing decentralization of government functions should facilitate this process.

### Workforce policy content and anticipated implementation

The respondents indicated ongoing challenges in recruitment policies in the past and were cautious regarding the expectations of new policy developments. There are three main government policy frameworks that should enable HWF development in Guinea. The first concerns the recruitment of HWF in public services [11, 12]. Although this policy has led to the actual recruitment of 3802 HWs in 2017, this study has not been able to obtain an actual annual costed plan to finance this expansion of the workforce. The national health financing strategy to attain universal health coverage (UHC) targets spending 15% of the total government budget on health by 2020. However, it lacks a plan to attain this increase and provides no insight into the proportion of the budget allocated to workforce development [26]. There is a considerable number of unemployed HWs away from regular health services [4]. While they are formally graduated, many have not received postgraduate training. Thoughtful planning and accompaniment will enable some of them to be integrated into the health system. It has been estimated that Guinea should increase its employed workforce by 17% annually to meet the standard it has set itself. While Guinea could be more ambitious in its scale-up of the workforce, it seems possible to make these investments within projected fiscal space available [27].

Secondly, the establishment of the ANSS aims to increase public HWF capacity for essential public health functions. In 2017, the ANSS contracted and deployed 10 HWs per district to increase this core capacity. Part of this staff was later integrated into the recruited personnel by the MoH. It remains unclear whether ANSS can still contract staff outside the wage bill in the future.

Thirdly, in the wake of the EVD epidemic, there is recognition that health issues must be addressed directly at the community level. The ministry, supported by organizations like UNICEF, has begun to develop a community health workforce (CHWF) to serve as an interface and hence increase trust between formal health services and communities [28]. The government was inspired by the Health Extension Program in Ethiopia, where a cross-country community-based program was rolled out [29]. The future capacity to actually manage an increased HWF in the districts, including their performance and career development, will be a challenge. Managing and supervising such a mixed HWF requires specific competencies, resources, and training. Continuous training possibilities and peer support are necessary to retain staff in remote posts. Respondents also suggest a system of rotation and scholarship schemes (for future specialization) to incentivize HWs to stay in rural areas [25].

### Actors, positions, and collaborations on HRH policy

The response to the EVD epidemic facilitated the emergence and presence of many “new” actors, both nationally and internationally. ANSS, NGOs, and international organizations have been recruiting personnel at the district level creating a new staff “mix.” The interviews indicate that there are challenges in aligning the workforce along common objectives. A first challenge is generational since districts have an existing group of often elder personnel that is now confronted with a new generation of younger staff, trained in Conakry, perhaps even with better remuneration. The position of the “volunteers,” basically unemployed HWs, in the health centers, requires similar attention. The results clarify that this cadre feels replaced by staff coming from outside while not being given a chance themselves to become civil servants. Both situations might create potential conflict situations that should be anticipated.

A second challenge is skills mix. While there are proportionally more medical doctors in the workforce, international policies and donors urge for task-shifting and the development of midwives and nurses [30]. Some international agencies mentioned that they focus on re-training and upgrading competences of ATS to have a proper midwife or nurse accreditation. This creates a new division of competences and tasks between the HWF and requires a clear framework on how primary healthcare teams function. A third challenge is possible tension between the village health teams initially engaged in addressing the Ebola outbreak and the CHWF deployed by organizations afterwards. At the central level, the MoH has attempted to create a terms of reference for the CHWF profile [22]. At the local level, there seems to be considerable variation in competences, remuneration, and profiles in the CHWF. The fourth challenge is related to remuneration and contracting. Several development actors said that they have the flexibility to recruit HWs on a short-term basis, often offering considerably higher salaries than the MoH. The remuneration differences and agreement on priorities require a strong district management team to coordinate different actors and programs. Nevertheless, workforce projections suggest that far more international support is needed in Guinea, Sierra-Leone, and Liberia to address their current training capacity weaknesses if acceptable levels of HWF are to be produced. A suitable investment on the part of “an international community awakened to the global security threat” [31] would be in supporting a significant scale-up of this capacity [27].

### Educational developments concerning the HWF

Interviewed actors acknowledged that there is no formal coordination matching the growing supply of graduated HWs in the more remote regions (Fig. 3) with labor market dynamics. The prospect of having a job in healthcare services might be reasonable in the long term but the short-term prospects for newly graduated students remain limited. Private schools focus mainly on nursing and midwifery training. The directors indicate that the bottleneck is the actual availability of competent teachers, internship possibilities to enhance practical skills, as well as limited accreditation and supervision. Exposure to practical healthcare work is limited, impeding graduate quality and potentially impacting population trust. Capacity development in the areas of HWF management, professional education, institutional development, and leadership requires attention and investment [32, 33]. The labor market mismatch requires reflection on student selection, management, and actually capping the number of entrance candidates [4]. The medical faculty of Gamal Abdel Nasser University has already imposed a student stop in 2016 to address the relative oversupply of medical doctors.

### Governance of the Guinean HWF

HWF governance is a complex health system function, bringing state and non-state actors as well as different sectors together at multiple levels. The centralized management lines do not easily facilitate such a “horizontal” approach. The respondents confirmed that in 2016, a new MoH-led HWF committee was established at the national level. This committee should technically be the governing body that analyses, oversees, plans, budgets, and evaluates several HWF policy actions. Ideally, all actors should buy-in to such a governance mechanism. An HWF committee could consist of representatives from several ministries, including the MoF. Workforce development should be aligned with fiscal and budgetary space to invest in the health sector. Although some international actors favored more sector-wide budget support, they demanded accompanying institutional reform of the MoH to rationalize health system development and improve efficiency and cooperation with other institutional government partners [13].

A good national HWF strategy and implementation plan should be based on evidence-based policy, labor market, and demographic needs analysis, and include a proper budget, monitoring and evaluation plan. Some countries including Sudan and Indonesia have developed an observatory to monitor HWF trends [34]. This requires capacity and policy space at the MoH to plan, manage, cost, and follow-up all actors adhering to one HWF plan [35]. Leadership must acknowledge that HWF is an important development (and not only health) issue, so seeking alignment and support from the main political and societal actors is required, even involving them actively in the HWF committee [35].

### HWF development in Guinea

The policy processes required to reform and develop the Guinean HWF in a sustainable matter is complex, using a range of interventions rather than a single-policy solution. A comparative study on HWF policies in four post-crisis settings indicated that these moments enable windows of opportunity for change and reform can occur but are by no means guaranteed—rather they depend on a constellation of leadership, financing, and capacity [36].

Two main recommendations can be provided based on the research. The first is that it is essential that there is guidance, commissioned research, and space for policy adjustments by the MoH on the implementation of the different HWF processes in the country, most notably the deployment of HWF to rural areas. This could improve the fragile trust between the government, HWF, and communities.

The second recommendation concerns the need to initiate dialogue with all relevant national-level actors to provide a situational and comprehensive labor market analysis of the HW situation, as to expand professional education, financing, and various trends in the country [19]. There is also a need to construct an HRH governance mechanism with its terms of reference and division of tasks of relevant actors involved decided.

### Limitations

Some difficulties were encountered during data collection. Firstly, two educational centers could not be visited, so data concerning these establishments were not collected. Only HWF supply and needs have been assessed in this study, while the financial and demand aspects require a different, in-depth analysis. Secondly, two key resource persons were not available for interviews. This limited accessibility to the latest government health budget and its breakdown for the HWF. Similarly, it has been impossible to assess the financial contribution of development partners to the budget for supporting staff recruitment. Finally, the participation of a foreign researcher conducting interviews could influence responses and possibly constituted a bias for the study.

## Conclusion

Health workforce development in Guinea requires a reform notably as there is a considerable mal-distribution of HWF between rural and urban areas. The weak state of the health system aggravated the EVD outbreak and led the government to initiate a plan to revitalize the health system and workforce. HWs were recruited in 2017 and deployed to rural areas for a minimum of 5 years. This study raises questions and challenges in terms of anticipated policy implementation, governance, HWF labor dynamics, and professional education aiming to achieve sustainable staff retention in rural areas. A longitudinal follow-up of this deployment will be undertaken to understand the structural issue driving the policy agenda including options related to staff retention and to evaluate the policy implementation of future medicalization of primary care in the districts and impact on the quality of services and health outcomes. The EVD outbreak provided for a policy momentum to reform the HWF in Guinea. All actors involved share a responsibility to sustain that momentum and strengthen the health system.

## Data Availability

The datasets used and/or analyzed during the current study are available from the corresponding author on reasonable request.

## Abbreviations

ANSS: National Health and Safety Authority (Agence Nationale de Sécurité Sanitaire)
ATS: Nurse assistants (Agents Technique de Santé)
CHWF: Community health workforce
CNFRSR: Centre National de Formation et de Recherche en Santé Rurale de Maferinyah
EVD: Ebola virus disease
HRH: Human Resources for Health
HW: Health worker
HWF: Health workforce
ITM: Institute of Tropical Medicine Antwerp
M&E: Monitoring and evaluation
MoF: Ministry of Finance
MoH: Ministry of Health
NGO: Non-governmental organization
SDG: Sustainable Development Goals
UHC: Universal health coverage
UN: United Nations
UNFPA: United Nations Population Fund
WHO: World Health Organization

## Footnotes

1 SDG Target 3c “Substantially increase health financing and the recruitment, development, training and retention of the health workforce in developing countries, especially in least developed countries and small-island developing States”

2 This target is set at 4.45 health workers/1000 population and is almost twice the first estimation made by WHO in 2006 (2.28).

3 Absenteeism means “a situation in which people are not at school or work when they should be”. Retention means “the ability of an employer to keep its employees and stop them from going to work somewhere else”. (Cambridge dictionary)

4 Comité national d’éthique pour la recherche en santé (CNERS). No. 130/CNERS/16. Guinée, Conakry, October 11, 2016

5 In 2016, “An SDG index threshold” of 4.45 doctors, nurses, and midwives per 1000 population was identified by the WHO as an indicative minimum density representing the need for health workers.
